# What Is the Role of Gut Microbiota in Obesity Prevalence? A Few Words about Gut Microbiota and Its Association with Obesity and Related Diseases

**DOI:** 10.3390/microorganisms10010052

**Published:** 2021-12-27

**Authors:** Julita Tokarek, Joanna Gadzinowska, Ewelina Młynarska, Beata Franczyk, Jacek Rysz

**Affiliations:** Department of Nephrology, Hypertension and Family Medicine, Medical University of Lodz, ul. Żeromskiego 113, 90-549 Lodz, Poland; julita.tokarek@stud.umed.lodz.pl (J.T.); joanna.gadzinowska@stud.umed.lodz.pl (J.G.); bfranczyk-skora@wp.pl (B.F.); jacek.rysz@umed.lodz.pl (J.R.)

**Keywords:** obesity, microbiota, dysbiosis, morbidities, gastrointestinal microbiome, probiotics, metabolic syndrome

## Abstract

Obesity is becoming the most dangerous lifestyle disease of our time, and its effects are already being observed in both developed and developing countries. The aim of this study was to investigate the impact of gut microbiota on the prevalence of obesity and associated morbidities, taking into consideration underlying molecular mechanisms. In addition to exploring the relationship between obesity and fecal microorganisms with their metabolites, the study also focused on the factors that would be able to stimulate growth and remodeling of microbiota. Assessed articles were carefully classified according to a predetermined criterion and were critically appraised and used as a basis for conclusions. The considered articles and reviews acknowledge that intestinal microbiota forms a multifunctional system that might significantly affect human homeostasis. It has been proved that alterations in the gut microbiota are found in obese and metabolically diseased patients. The imbalance of microbiome composition, such as changes in Bacteroidetes/Firmicutes ratio and presence of different species of genus *Lactobacillus,* might promote obesity and comorbidities (type 2 diabetes mellitus, hypertension, dyslipidemia, depression, obstructive sleep apnea). However, there are also studies that contradict this theory. Therefore, further well-designed studies are needed to improve the knowledge about the influence of microbiota, its metabolites, and probiotics on obesity.

## 1. Introduction

### 1.1. Obesity Epidemic—Statistics, General Background, Causes and Effects

Obesity is now recognized as a global epidemic affecting both developed and poor-resource countries [1], which lowers life expectancy [2] and has extensive consequences for countries’ health care systems [1]. Body mass index (BMI), which is weight in kilograms divided by height in meters squared, is used to identify obesity. For adults, a BMI of 25.0 to 29.9 kg/m^2^ is defined as overweight and a BMI of 30 kg/m^2^ or higher is defined as obese [3]. In the last three decades, the worldwide prevalence of obesity has increased 27.5% for adults and 47.1% for children [3]. By 2030, 81% of men and 74.9% of women in the USA are projected to be obese or overweight, whereas it is expected that by 2030, approximately 39% of children and 46% of adolescents will have an abnormally high BMI [4]. Childhood obesity (defined as body mass index-for-age (or BMI-for-age) percentile greater than 95 percent) is an important risk factor for adult obesity: a meta-analysis of available data showed that half of obese children were still obese as adults; additionally, risk is more than twofold if both parents are obese [5].

The etiology of obesity is multifactorial and includes genetic, hormonal, socioeconomic, environmental, and cultural influences [3]. Comorbidities and their treatment might be a factor in the prevalence and progression of obesity as well. Primary and secondary disease-related causes of obesity are presented in Table 1. 

The primary causes of obesity can be classified as being related to genetic disorders in the form of monogenic diseases and genetic syndromes [6]. Monogenic obesity is a rare and severe early-onset obesity inherited in a Mendelian pattern with abnormal feeding behavior and endocrine abnormalities. It is mainly caused by autosomal recessive mutations in genes of leptin, pro-opiomelanocortin (POMC), pro-hormone convertase 1, and melanocortin 4 receptor (MC4R), which play a key role in the hypothalamic control of food intake. The predominant features of the genetic syndromes associated with obesity presented in Table 1 are physical characteristics, including dysmorphic features, developmental delay, and mental retardation. Existing genetic defects are often chromosomal abnormalities that usually involve multiple genes. Prader–Willi syndrome is one of the most common syndromic forms of obesity in children [6]. 

Secondary causes of obesity include diseases involving systemic dysfunctions and disorders of regulatory mechanisms causing metabolic changes in the body, which promote the development of obesity secondary to the primary disease. Disturbances in homeostasis resulting from diseases lead to the body’s inability to maintain energy balance, dysregulation in hormone synthesis and secretion, abnormal energy expenditure, and altered consumption behavior [7].

The association between gestational weight gain and obesity has been investigated, and maternal gestational weight increase is an independent predictor of obesity in infancy [8]. There is a strong association of genetics with obesity, which means that the involvement of multiple genes and their complex interaction can result in the manifestation of the disease, which can be a monogenic (5% of the cases) or a polygenic obesity type [9]. Obesity treatment consists of bariatric surgery and medical treatment, of which undergoing a surgery provides a longer life expectancy [10].

### 1.2. Association of Obesity with Other Diseases

Obesity increases the risk of other associated diseases, as it has been recognized as a key factor inhibiting DNA damage repair mechanisms. Cellular response to DNA damage can result in irreversible cell-cycle arrest, activation of several proteins that can induce adipocyte differentiation and hypertrophy, disturbances in cell metabolism, impairment of glucose metabolism, and promotion of the development of systemic insulin resistance [11]. The most well-established weight-related comorbidities are insulin resistance, type 2 diabetes, and cardiovascular disease, the risks of which are proportional to BMI [12]. Moreover, the obesity is linked with various immediate and long-term adverse health outcomes such as sleep apnea, hypertension, heart disease, stroke, osteoarthritis, and certain types of cancer and leads to psychosocial problems such as stigmatization and poor self-esteem [13].

The adverse effects of obesity on cardiometabolic health are indisputable; abdominal obesity is especially significant in the pathogenesis of cardiovascular disease and leads to well-identified disturbances in adipocyte biology and adipose tissue inflammation with direct systemic metabolic consequences such as endothelial dysfunction and atherogenesis [14].

Furthermore, multiple studies reveal a strong association between COVID-19 and obesity. COVID-19 patients with obesity have an enhanced hospitalization rate, more severe progression, and worse clinical outcomes [15].

Comorbidities of obesity are presented in Table 2.

### 1.3. Unhealthy Diet and Lifestyle and Their Relationship to Obesity

It is commonly stated that urbanization is one of the most important drivers of the worldwide rise in BMI because diet and lifestyle in cities lead to adiposity; however, in high-income and industrialized countries, a persistently higher rural BMI was noted, especially for women [19]. Sedentary behavior and time spent in front of TV are both related to a higher odds of obesity [20]. Among university students, being male, the family home not being in the university city, having a mother of low socioeconomic status, and finally, not studying a health-related course are the factors associated with a lower quality diet [21]. A positive correlation was found between BMI and consumption of a Westernized and high in protein/fat diet [22].

Studies conducted on adults showed that experimental reduction in sleep duration downregulates the satiety hormone, leptin, and upregulates the appetite-stimulating hormone, ghrelin, and increases hunger and appetite [23].

### 1.4. Gut Microbiome and Obesity

The body’s microbiome, bacteria, viruses, archaea, and eukaryotic microbes residing in and on the body have the potential to impact our physiology in several ways, including contributing to metabolic function. Studies have demonstrated that the composition of gut microbiome can among many other functions increase dietary energy intake, and therefore, promote the obese phenotype [3]. 

Throughout the gastrointestinal tract, differences in the composition of the microbial population have been observed. Due to the high motility of the esophagus and the acidic unfavorable environment of the stomach, there are quantitatively the fewest microorganisms, and the predominant bacteria come from the oral cavity (e.g., *Streptococci* and *Lactobacilli*). In the intestinal microbiota, there are already many more bacteria, e.g., in the initial part of the jejunum, the most numerous is the genus *Streptococcus*, while the ileocecal region is inhabited by the subgroup *Bacillus* bacteria (phylum Firmicutes, mainly Streptococcaceae), bacteria of the phylum Actinobacteria (especially the subgroups Actinomycinaeae and Corynebacteriaceae), Bacteroidetes, and Lachnospiraceae. The largest number of bacteria and the greatest microbial diversity are found in the distal segment of the ileum and colon because there is a more favorable pH for bacterial colonization, and in addition, due to antiperistaltic contractions, the food content is retained longer in the intestinal lumen. There are mainly the Gram-positive bacteria *Bacteroides* and *Clostridium*, *Lactobacillus*, *Enterococcus*, and Enterobacteriaceae. 

The relationship between gut microbiota imbalance and obesity has been widely studied and commented on recently, as the influence of some substances produced by the microbiota and several molecular pathways on the development of obesity has been demonstrated. However, the correlations remain very complex, and therefore, only selected ones will be discussed in the following sections. The results discussed here indicate the possibility of manipulating the gut microbiota in the therapy or prevention of obesity and its metabolic consequences [24]. 

## 2. Function and Physiology of Adipose Tissue

Adipose tissue is a compound and highly active metabolic and endocrine organ that consists of adipocytes, connective tissue, nerve tissue, and immune cells. These components together form an integrated and multifunctional unit [25].

There are two main types of adipose tissue distinguished in human body—white adipose tissue (WAT) and brown adipose tissue (BAT) [26]. Their content in the organism depends on genetic, metabolic, and environmental factors, but WAT makes up the majority of adipose tissue in adults [27].

### 2.1. Morphology

The differences between adipocytes in WAT and BAT can be observed via light microscope.

White adipocytes are spherical cells with a diameter of 10–100 μm [27]. Their organelles along with the compressed nucleus are in the perimeter of the cell due to a unilocular lipid droplet that occupies a major part of the cytosol [28].

Brown adipocytes typically present an ellipsoid shape that ranges from 15 to 50 μm in diameter and contains multilocular lipid droplets [27]. The nucleus of these cells is in the central part of the cytoplasm. BAT is also characterized by the expression of the proton transporter UCP (uncoupling protein) [28].

### 2.2. Function and Physiology

The adipose tissue is characterized by high plasticity and the ability to change volume as well as composition based on the energetic status of the body. An increase in the weight of adipose tissue may occur in both hypertrophy and hyperplasia [29].

The most important function of WAT is storage of triglycerides during increased energy supply and exploitation of these reserves during higher energy expenditure, while BAT plays a vital role in the process of thermogenesis, especially in infants [29].

Despite the differences in functions, both WAT and BAT present endocrine activity [30]. The factors secreted by the adipose tissue include leptin, adiponectin, complement components, plasminogen activator inhibitor-1, proteins of the renin–angiotensin system, and resistin. Moreover, metabolism of sex steroid hormones and glucocorticoids also takes place in adipocytes [25].

### 2.3. Endocrine Activity

Leptin was initially described as an anti-obesity hormone; however, current studies have proved that its function concentrates on energy sufficiency rather than on excess [31]. Therefore, lower energy intake results in a decrease in leptin levels, which is associated with physiological responses to starvation, such as increased appetite and restricted energy expenditure. This function complies with the evolutionary mechanism that prevents energy sufficiency but is not needed in current highly developed societies [25]. Furthermore, many forms of obesity result in elevated circulating leptin. However, both endo- and exogenous high leptin levels do not act against developing obesity. The mechanism for the occurrence of leptin resistance is unknown, but it may be a result of defective leptin signaling or transport across the blood–brain barrier [32].

Both adiponectin and resistin participate in the mechanism of insulin resistance.

There is an inverse association between adiponectin levels and insulin resistance—adiponectin levels are low with insulin resistance, and treatment with adiponectin may improve metabolic parameters in patients with obesity or lipodystrophy. Consequently, the increase in adiponectin levels occurs when insulin sensitivity improves [33].

On the other hand, resistin belongs to molecules that impair insulin-stimulated glucose uptake, which may lead to insulin resistance [34]. However, many epidemiological studies did not provide a valid link between resistin expression in adipose tissue or circulating resistin levels and the occurrence of this condition [35].

## 3. Function of Gut Microbiota

Gut microbiota consists of about 80 trillion bacteria that create a complex system that plays a vital role in maintenance of homeostasis in human organism. This includes, e.g., regulation of the host’s metabolism, provision of proper function of the intestinal barrier, and immunomodulation [36]. More than 100 bacterial species could be found in the intestines, and there are about ten times more bacterial cells present in gut microbiota than human cells in the body [37]. Most bacteria that form the gut microbiota belong to four bacterial phylotypes: Bacteroidetes, Firmicutes, Proteobacteria, and Actinobacteria, mainly anaerobic species [38,39]. However, various kinds of virus, protozoa, archaea, and fungi could also be a part of intestinal microflora [36].

The development of gut microbiota begins immediately at birth—the fetus is exposed to a bacterial population for the first time during passage through the birth canal [40]. That is the reason why infants’ microbiota can consist of similar bacteria that form the vaginal microbiota of their mothers [41]. Moreover, there is a proven difference in the composition of gut microbiota among infants delivered through caesarean section and those delivered vaginally [42].

The composition of intestinal microbiota undergoes many changes during a lifetime; nevertheless, the microbiota of one-year-old children tends to stabilize and begins to resemble that of young adults [41]. Due to this fact, the initial colonization of the gastrointestinal tract might be an important factor determining the composition of the microbiota in adulthood [40]. Studies on mono- and dizygotic twins have even proved that the bacterial colonization during birth had more impact on their adult microbiota than genetic factors [43]. However, the development of gut microbiota is a complex process and depends on many different factors (e.g., diet, usage of probiotics or antibiotics) and further studies are necessary to fully establish the parental role in determining the composition of the adult microbiota [40].

**Figure 1:**
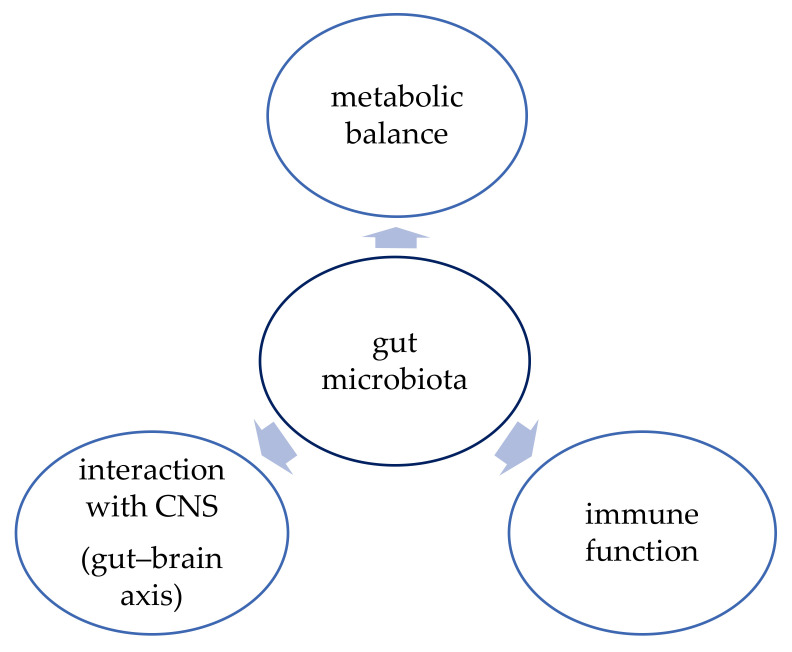
Function of gut microbiota [44].

The function of human gut microbiota has been an object of interest of many studies conducted in the last few years. The function of the gut microbiota is shown in Figure 1. The microbiota proved to have a vital role in maintaining metabolic balance and proper function of the immune system, but there is also evidence that it can influence brain development and neurogenesis and that it interacts with the central nervous system (CNS) via the “gut–brain axis” [44]. Therefore, better understanding of this complex system might bring promising therapeutic options not only for obesity but also for depression, inflammatory bowel diseases (IBD), or cancer, for example [37].

### 3.1. Metabolic Balance

The bacteria present in the intestines create an extraordinary metabolic “organ” that can extract nutrients and energy from ingested food. For example, the gut microbiota enables the catabolism of dietary fiber that cannot be fully hydrolyzed by human enzymes during digestion. Short chain fatty acids (SCFAs) are the main product of this process, and they consist of fatty acids with fewer than six carbons [38]. Different studies proved that SCFAs could influence appetite regulation and the metabolism of lipids and glucose [45]. These molecules could also affect the integrity of the intestinal barrier by promoting the proper function of tight junctions (TJs) between epithelial cells and hence regulating the absorption of xenobiotics [38].

### 3.2. Immune Function

The gut microbiota contributes to the proper function of the immune system in two different ways [46]. First, it provides physical protection against enteropathogens by maintaining the structural integrity of the intestinal barrier [38]. Second, the intestinal microbiota plays an important role in immunomodulation. Symbiotic bacteria can influence the immune response of the host by improving the activity of macrophages and natural killer (NK) cells [46]. They also promote tolerogenic dendritic cells and regulate inflammatory-related pathways [38].

### 3.3. Gut–Brain Axis

The mechanism in which the bidirectional communication between the gut microbiota and the brain occurs has not been fully explained yet. However, the studies have shown there are many ways that allow this pathway to function, including neuroanatomical pathway and the endocrine, immune, and metabolic system [36]. This network allows the brain to affect gut movement and modify the sensory and secretion function of the intestines [36]. On the other hand, microorganisms present in the gut microbiota can produce neurotransmitters (e.g., dopamine) [44] and promote the release of gut hormones from gut enteroendocrine cells [47]. These transmitters provide the link to affect brain function [47].

## 4. Modification of Gut Microbiota

Many different factors relate to alterations in the composition of gut microbiota. Diet, age, use of antibiotics, and environmental factors belong to those of the highest importance [48].

### 4.1. Diet

The gut microbiota undergoes noticeable differences depending on the diet. Studies have shown that statistically important changes in the gut microbiota are induced by consuming a diet that varies in the amount of carbohydrates, fat, and fiber consumed [37,48]. Furthermore, the composition of microbiota differs among cultures, which might also correspond with differences in diet [37].

### 4.2. Age

Studies proved that age-related changes in the gastrointestinal environment together with lower immune system reactivity and increased medication use could change the composition of gut microbiota [37].

### 4.3. Antibiotics

The usage of antibiotics leads not only to the elimination of pathogenic microorganisms but also to the elimination of symbiotic microflora, which could result in a significant imbalance in gut microbiota [38]. The level of microbiota alteration depends on an antibiotic’s class, pharmacokinetics, pharmacodynamics, and range of action, as well as dosage, duration of treatment, and administration route [49]. Moreover, the antibiotics alter the composition of gut microbiota because of a direct harmful effect on the gastrointestinal epithelium and the spread of antibiotic-resistant bacteria [49].

### 4.4. Probiotics

What is more, intake of dietary supplements containing probiotics and prebiotics could be another factor that might have an impact on gut microbiota [37]. According to WHO, probiotics are defined as “live micro-organisms, which when consumed in adequate amounts, confer a health effect on the host” [50]. The most frequently used bacterial genera are *Lactobacillus* and *Bifidobacterium* [51]. There are at least four mechanisms in which probiotics could have a positive effect on a host’s intestines [51].

1. Probiotics present the ability to modify the composition of microbiota. They contain bacteria that can produce inhibition bacteriocins, biosurfactants, and SCFAs that together form an environment that is more favorable for the growth of symbiotic microorganisms and a reduction in the colonization of pathogenic bacteria [51].
2. Probiotics play an important role in the improvement of the intestinal barrier by preventing the proper function of tight junctions between epithelial cells. Moreover, they can stimulate the production of antimicrobial peptides (e.g., defensins) in gut endocrine cells [51].
3. Probiotics might function as modulators of the immune system. They can act through gut-associated lymphoid tissue (GALT). The interaction between pattern recognition receptors (PRRs) and microbial-associated molecular patterns (MAMPs) present on the surface of microorganisms results in activation of immune cells and production of proper cytokines. The effect of activation of the immune system might be both local and systemic, depending on the severity of the stimuli [51].
4. Probiotics provide enzymes that cannot be produced by the host’s organism (e.g., beta-galactosidase), and therefore they complement the process of digestion [51].

## 5. Microbiota and Obesity

Alterations in the composition of the human gut microbiota occur in metabolic disorders such as obesity, diabetes, and eating disorders, as well as in stress-related neuropsychiatric disorders including depression and anxiety [52]. Some studies found that when using human colonic tissue, microbiota encroach within the gut wall, and the degree of encroachment directly correlated with dysglycemia in adults [53]. Some mechanisms have been proposed to explain the role of gut microbiota in obesity development.

### 5.1. Short Chain Fatty Acids

The gut microbiota is responsible for metabolizing energy from the diet, e.g., indigestible dietary fibers, which are chemically polysaccharides and oligosaccharides. They are converted into short chain fatty acids (SCFA) that are either absorbed by the gut or excreted in feces, such as acetate, propionate, and butyrate. After absorption, SCFAs can induce lipogenesis and increase triglyceride stores through molecular pathways. They activate the carbohydrate-responsive element-binding protein and the sterol regulatory element-binding transcription factor 1, both involved in the process of lipogenesis. The rate of SCFA metabolism can establish the direction of host energy balance by increasing the effectivity of calorie uptake. Moreover, SCFAs suppress the fasting-induced adipocyte factor, which inhibits lipoprotein lipase, inducing triglycerides accumulation in adipocytes [54]. Dietary fibers, which are the main source of SCFAs, are suggested to have a protective effect for maintaining a healthy body weight. Several mechanisms have been suggested for how SCFAs may participate in the development of obesity: (1) SCFAs provide an extra energy source (approximately 10% of daily energy requirement) and contribute to extra fat deposition in the body; (2) SCFAs are ligands for G protein-coupled receptor GPR 41 and GPR43, which are expressed in the intestinal epithelium, immune cells, and adipocytes and are responsible for regulating energy expenditure; (3) regulation of fasting-induced adipose factor (FIAF); (4) de novo lipogenesis; (5) regulation of glucose homeostasis; (6) regulation of leptin secretion via free fatty acid receptor (FFAR); and (7) modulation of satiety response [55]. In the liver, SCFAs have miscellaneous actions: propionate is gluconeogenic, whereas acetate and butyrate are lipogenic; however, results from studies are inconsistent. Propionate promotes intestinal lipolysis and energy homeostasis in mice through the AMPK/LSD1 pathway. Butyrate is the colon’s main energy source, and intestinal epithelial cells derive most of their energy from the oxidation of butyrate. The increase in butyrate-producing bacteria in the gut microbiota and increased production of butyrate thereby improves lipid metabolism through the butyrate–SESN2/CRTC2 pathway. However, excessive butyrate may reduce the proportion of probiotics and reverse the metabolic effects [56].

There are also methanogenic Archaea (represented by *Methanobrevibacter smithii*) that oxidize H_2_ produced by other bacteria by chemical reaction with carbon dioxide (CO_2_). In animal models, this process was associated with higher extraction of polysaccharides from the diet by the removal of H_2_ formed by fermentation and increased production of short chain fatty acids (SCFA) [54]. This process not only removes excess H_2_ from the intestine, which compromises the fermentation process, but also causes a reduction in H_2_ with CO_2_ for methane production, leading to better use of a hypocaloric diet [57]. Germ-free mice weigh less and lose twice as many calories in their stool and urine compared with conventional mice on the same diet, thus supporting the notion that the gut microbiota enable the host to extract more energy from the food source and use it toward host energy balance and weight maintenance. Whether the role of SCFAs in host metabolism is positive versus negative remains to be seen, given contradicting evidence [58].

Increasing evidence suggests the stimulating effects of SCFAs supplementation on incretin and other gut hormones, which include glucose-dependent insulinotropic polypeptide (GIP), peptide YY (PYY), and glucagon-like peptide-1 and 2 (GLP-1 and GLP-2). They are involved in regulating gut motility, satiety, and glucose metabolism [52]. In mice with diet-induced obesity, SCFA supplementation improved insulin resistance and obesity. In other animal studies, butyrate-producing bacteria, such as *F. prausnitzii*, have appeared to alleviate insulin resistance by inducing glucagon-like peptide 1 (GLP-1) secretion from the colonic L cells by signaling through the fatty acid receptor FFAR2. Another mechanism through which butyrate and propionate may influence host glucose metabolism is the activation of intestinal gluconeogenesis [58].

In recent years, SCFAs have attracted special attention due to their beneficial effects on intestinal homeostasis and energy metabolism, but their role in obesity remains controversial. Higher concentrations of fecal SCFAs are associated with gut permeability, metabolic disorder markers, obesity, and hypertension. Although they can protect the host from diet-induced obesity, excessive SCFAs can provide extra energy for the host, thus promoting obesity [56].

### 5.2. AMPK and Fiaf

Gut microbiota can decrease liver fatty acid oxidation by suppressing adenosine monophosphate kinase (AMPK) and fasting-induced adipose factor (Fiaf), a circulating lipoprotein lipase inhibitor, which as a consequence causes increased fat accumulation [52]. The bacterial suppression of the expression of Fiaf and AMPK in the liver and skeletal muscle, leads to weight gain from a carbohydrate and fat rich diet [59]. 

Fiaf is produced by the intestine, liver, and adipose tissue. Inhibition of Fiaf results in increased activity of LPL, which mediates cellular uptake of triglycerides and accumulation of triglycerides in adipocytes. Germ-free mice that were populated with microbiota throughout the study showed suppression of Fiaf expression in intestinal epithelial cells. After conventionalization, Fiaf-/- germ-free mice had 57% more total body fat than their wild-type counterparts, and furthermore, they were not protected from diet-induced obesity. *Bacteroides* and *Clostridium* were the most prevalent types of bacteria found in the digestive tract of conventionalized mice, as examined by sequence-based 16S rDNA enumeration studies. Regarding the modulation of lipid metabolism by colonization with single saccharolytic bacterial species, *Bacteroides thetaiotaomicron* has been shown to play an important role. In germ-free mice, colonization by *B. thetaiotaomicron* altered the expression of host genes involved in lipid degradation and absorption and polysaccharide metabolism [60]. 

Colonization with a single bacterial species showed a statistically significant increase in total body fat, although the magnitude of the increase was less than that obtained with unfractionated gut microbiota. Interestingly, in another study, mice fed a high-fat diet supplemented with *Lactobacillus paracasei* showed a reduction in body fat and an accompanying increase in Fiaf activity [61].

It has also been shown that germ-free mice present increased levels of phosphorylated AMPK in muscle and liver, which protects them from diet-induced obesity. AMPK stimulates fatty acid oxidation in peripheral tissues by activating mitochondrial fatty acid oxidation enzymes such as acetyl-CoA carboxylase and carnitine-palmitoyltransferase I. However, a decrease in AMPK and adiponectin activity was observed in normal and obese mice, along with a decrease in fatty acid oxidation of fatty acids and an increase in the influx of free fatty acids into the liver [62]. 

### 5.3. Bile Acids

The gut microbiota changes the composition and relative abundance of bile acid species, which might explain its effect on glucose and insulin homoeostasis. A reduced bile acid concentration in the gut has been associated with bacterial overgrowth and inflammation [52]. Recent studies suggest FXR signaling as an important pathway for the interaction between the gut microbiota and bile acids. For example, studies in human and murine models showed a change in the gut microbiota post-RYGB (Roux-en-Y gastric bypass) toward a pattern associated with thin phenotype, including a decrease in *Bacteroides* and an increase in *Roseburia*. One pathway within FXR signaling through which bile acids and gut microbiota contribute to host metabolism is by the gut microbiota metabolizing bile acids into primary and secondary bile acids, which then bind to the FXR receptor and stimulate secretion of gut-derived hormones, such as fibroblast growth factor FGF-19. In turn, FGF-19 regulates bile acid synthesis as well as lipid and glucose metabolism. Increased bile acid synthesis contributes to increased energy expenditure in the host by stimulating the brown adipose tissue and skeletal muscle via TGR5, a membrane bound G protein-coupled bile acid receptor, and by increasing thyroid hormones by activating type 2 deiodinase [58]. Secondary bile acids bound to cellular receptors, such as TGR5, reduce macrophage inflammation and lipoprotein uptake, resulting in less atherosclerotic plaque formation, which decreases the development of atherosclerosis [63].

### 5.4. LPS

Lipopolysaccharides (LPS) contain lipid A, which can cross the intestinal mucosa through tight junctions or with the aid of chylomicrons. Lipoproteins are responsible for the absorption and transport of dietary triglycerides and could thus initiate an inflammatory process that could result in the insulin resistance often observed in obesity. LPS concentrations are low in healthy people but may reach high concentrations in obese individuals and cause metabolic endotoxemia. Metabolic endotoxemia increases adipocyte hyperplasia and recruitment of macrophages into adipose tissue in a CD14-dependent pathway and increases the production of activin A, which activated the proliferation of adipocyte precursor cells [63]. Gut microbiota also may contribute to metabolic disturbances observed in obese patients by triggering systemic inflammation. Cell membrane LPSs of the Gram-negative bacteria from gut microbiota bind toll-like receptors (TLRs), mainly TLR4, which are immune transmembrane proteins. They can upregulate inflammatory cytokines and chemokines and engage intracellular signaling pathways, regulating the nature, magnitude, and duration of inflammatory response [52]. The binding of LPS to the TLR4 receptor initiates a cascade of inflammatory events with the secretion of proinflammatory cytokines such as interleukin 6 (IL-6) and tumor necrosis factor a (TNF-a) [57]. Therefore, LPS-mediated endotoxemia affects brain inflammation by enhancing the passage of circulating inflammatory cytokines across the blood–brain barrier, stimulating microglia via TLR4, and/or inhibiting vagal afferent neurons. Moreover, LPS triggers prostaglandin-endoperoxide synthase 2/cyclooxygenase 2 (Ptgs2/COX2) mRNA expression and consequent higher levels/activity of COX2, a pro-inflammatory enzyme that catalyzes the conversion of arachidonic acid to prostanoids [64]. Acute intravenous injection of LPS (2.5 mg/kg) in mice induces a morphological transition from resting microglia into activated and round macrophage-like cells in the hypothalamus, thalamus, and brainstem starting from ~8 h to 24 h after injection, with complete recovery at 7 days after injection. In HFD (high-fat diet) obese mice, hypothalamic microglia undergo a biphasic activation showing a first inflammatory reaction at 2 weeks, followed by a reduction in inflammatory markers, which return to higher levels after 4 weeks when bone marrow-derived myeloid cells gradually replace these cells [64].

Studies have shown that increased levels of butyrate-producing Ruminococcaceae and Lachnospiraceae lead to reduced levels of members of the LPS family S247, thereby reducing chronic low-grade inflammation [56]. Furthermore, butyrate stimulated the expression of the transforming growth factor (TGF-β) from human intestinal epithelial cells, which, in turn, activated anti-inflammatory regulatory T cells. Similarly, *F. prausnitzii*, a butyrate producer, is thought to alleviate inflammation through its metabolites that block nuclear factor-Kb activation and subsequent secretion of proinflammatory mediators [58]. Overall, currently available evidence suggests that changes in the gut microbiota could contribute to the pathogenesis of obesity and to the development of obesity-related metabolic disorders, including type 2 diabetes, NAFLD, metabolic syndrome, and cardiovascular disease. Obesity treatments such as calorie reduced diets and/or bariatric surgery modify the gut microbiota in ways that are associated with health benefits, supporting the hypothesis that changing gut microbiota composition has the potential to provide an additional mechanism for achieving stable weight loss [65].

## 6. Obesity, Genetics, and Environment

More than 50 phyla are known, but two are predominant and cover 90% of the bacterial population in both humans and mice. The first are Firmicutes (Gram-positive), which constitute 60–80% of the microbiota and include more than 200 genera (of which the most significant are *Ruminococcus*, *Clostridium*, and *Lactobacillus*). The second are Bacteroidetes (Gram-negative, including *Bacteroides*, *Prevotella*, and *Xylanibacter*), which make up 20–30% of the microbiota, followed by Actinobacteria (Gram-positive), which constitute around 10% of the microbiota (with a predominance of the genus *Bifidobacterium*) [66].

There is evidence for the association between gut bacteria and obesity both in infancy and in adults. The microbiome is a fingerprint of both the environment and human heritable genetic material. Ley et al. analyzed the gut microbiota of leptin-deficient mice at the major phyla level using the method of 16S rRNA gene sequencing in mouse models. The results showed how mice homozygous for an aberrant leptin gene ob/ob carried a different proportion of bacteria in the ceca compared with lean wild-type (+/+) or heterozygous (ob/+) mice. In particular, the ob/ob mice had a 50% decrease in the population of Bacteroidetes and a proportional increase in Firmicutes (*p* < 0.05).

Similarly, Turnbaugh et al. published a study on mouse models using the newer shotgun metagenomic sequencing technique on cecal microbial DNA (ob/ob, ob/+, and +/+). This study confirmed the increased ratio of Firmicutes versus Bacteroidetes in obese mice, as compared to lean ones. Moreover, ob/ob mice had a higher proportion of Archaea within the cecal gut microbial communities. Research in another mammalian model noticed a lower abundance of Bacteroidetes associated with obesity [24].

Both in humans and in animal models, obesity is correlated with changes in the Bacteroidetes/Firmicutes ratio, and obese individuals would present predominance of Firmicutes and a smaller proportion of Bacteroidetes when compared with non-obese.

In studies with humans, comparisons of the microbiota were made among 20 obese individuals, 20 healthy ones with normal weight (controls), and 9 patients with anorexia nervosa. The comparison confirmed the reduction in Bacterioidetes in obese people, as observed in animal models, and there was no difference for Firmicutes among the groups. It was observed, however, that there was a significant increase in *Lactobacillus* in the obese individuals. In the same study, the anorexic patients showed a significantly higher concentration of *Methanobrevibacter smithii*, suggesting a possible adaptive response to nutrient deprivation [57].

By contrast, some studies in humans have found an opposite ratio, suggesting that the Firmicutes-to-Bacteroidetes ratio is not a determinant in human obesity. Numerous researchers have studied how diet can modulate the Firmicutes-to-Bacteroidetes ratio. For example, the fecal microbiota of African children consuming a high-fiber diet showed a significant enrichment in Bacteroidetes and depletion in Firmicutes, with abundance of bacteria from *Prevotella* and *Xylanibacter* (Bacteroidetes) with respect to European children consuming a Western diet. On the other hand, Gram-negative bacteria such as *Shigella* and *Escherichia* were significantly underrepresented in African children and was relatively weak, and its detection was confounded by large interpersonal variation and insufficient sample sizes [55].

The studies in infants show that the vaginally delivered neonate is initially colonized with the vaginal and distal gut bacteria of the mother, while babies delivered by caesarean section (C-section) are initially colonized predominantly with the mother’s skin bacteria. More specifically, vaginally delivered neonates have relative and absolute larger populations of *Bacteroides* and *Bifidobacteria* species than those born by C-section. The observations that increased Bacteroidetes populations are present in both obesity and children born by C-section suggest that the infant microbiome may contribute to the subsequent ~40% increased risk of obesity in children and young adults delivered by C-section [54].

The genera *Staphylococcus* and *Clostridium* have been shown to be positively associated with obesity. A decrease in the genus *Faecalibacterium* was reported after LSG (laparoscopic sleeve gastrectomy), while the same genus increased after RYGB (Roux-en-Y gastric bypass). All these genera belong to the Firmicutes phylum [63].

Within the *Lactobacillus* genus, different species are associated with an obese profile (*Lactobacillus reuteri*) or a lean profile (*Lactobacillus gasseri* and *Lactobacillus plantarum*), such that the microbiota composition is related to body weight and obesity at the species level [67].

According to the studies, abundance of *Akkermansia muciniphila*, one of most widely studied gut bacterial species in relation to obesity, was lower in obese mice. Furthermore, increasing its intestinal abundance either with oligofructose or live culture gavage led to decreased endotoxemia, body fat, improved insulin sensitivity, and protected integrity of the gut barrier [68].

This lack of consensus on the relationships between the specific bacteria and obesity may be due to the different methods used for metagenomics sequencing, differences in sample microbial load, and insufficiency of looking at bacteria at the phylum level to fully understand the key bacterial members contributing to host obesity. In general, obese people have an unbalanced gut microbial community with a low diversity; therefore, it may be more effective to increase the balance and diversity of gut microbial community for preventing obesity than to feed or eliminate certain bacteria [69].

## 7. Interactions between Microbiota and Obesity

The studies have proved that there are certain bacterial genera associated with obesity. These microorganisms promote many different molecular patterns that lead together to the maintenance of obesity [63].

### 7.1. Immune System

The excessive amount of some Gram-negative bacterial species in patients with obesity interacts with the protein structure of endothelial “tight junctions”, which results in increased intestinal permeability. Furthermore, this intestinal dysbiosis accelerates the absorption of LPS (bacterial endotoxin) through CD14 receptor. Plasma LPS functions as a trigger factor of immune cascades that promote the production of pro-inflammatory cytokines. Subsequently, a pro-inflammatory phenotype is related to the maintenance of obesity [65].

### 7.2. Lipid Metabolism

The bacteria from the gut microbiome produce bile salt hydrolase (BSH) enzymes, which enable the production of unconjugated bile acids and secondary bile acids. These bile acids might bind to G protein-coupled receptor TGR5 and decrease pro-inflammatory phenotype and lipoprotein uptake. Moreover, the gut microbiota interacts with lipid metabolism because of the assimilation of cholesterol by bacterial cells and builds it into bacterial walls. These both patterns play a vital role in protection against the development of atherosclerosis and might be disrupted because of the change in composition of microbiota in obese patients [63].

What is more, due to increased activity of the gut endocannabinoid system through stimulation of endothelial CB1 receptors, unbalanced microbiota impairs the integrity of the intestinal epithelium and promotes metabolic endotoxemia. This process stimulates production of activin A, which activates the proliferation of adipose precursor cells and adipocytes hyperplasia [63].

### 7.3. Satiety Hormones

The gut microbiota can excrete molecules that stimulate enteroendocrine cells to produce the so-called satiety hormones. This mechanism is most likely associated with the secretion of propionate by commensal bacteria.

The composition of microbiota characteristic of patients with obesity presents the tendency to increase the production of ghrelin, which is associated with higher food intake. On the other hand, lower concentration of GLP-1 and PYY has been noticed in obese individuals. Both GLP-1 and PYY are secreted by intestinal L cells and control the feeling of satiety, which might regulate food intake. Furthermore, GLP-1 is an important factor stimulating the proper secretion of insulin. That is why the disturbances in GLP-1 concentration might lead to insulin resistance [63].

### 7.4. Nutrient Metabolism

The most important mechanism involving gut microbiota in the process of nutrient metabolism refers to the short chain fatty acids (SCFAs). The bacteria present in the intestines have the ability to secrete enzymes that take part in carbohydrate metabolism. They convert ingested dietary fibers into SCFAs, which are an extra source of energy that might eventually be accumulated as lipids or glucose [65].

### 7.5. Lymphoid Structures

The gut-associated lymphoid tissue (GALT) consists of Peyer’s patches, isolated lymphoid follicles, and crypt plaques. The gut microbiome interacts with the formation of GALT because the bacterial peptidoglycan can be recognized through pattern recognition receptors (PRR), nucleotide-binding oligomerization domain-containing protein 1 (NOD1), and Toll-like receptors (TLRs) that are present in the epithelial cells. Stimulation of these receptors increases the expression of CC chemokine ligand 20 (CCL20) and β defensin 3 ligand (HBD3). This leads to the formation of new isolated lymphoid follicles. Any disturbances of this mechanism due to the imbalance in the composition of gut microbiota might result in a pathologic inflammatory response in the gastrointestinal tract [63].

### 7.6. Microbiota–Adipose Tissue Axis

Metabolites of gut microbiota promote adipogenesis by triggering lipopolysaccharides (LPS)-based inflammation and short chain fatty acids (SCFAs)-induced adipocyte differentiation [70].

Lipopolysaccharides (LPS), the cell membrane component of Gram-negative bacteria, act as triggering factors leading to low-grade chronic inflammation followed by the development of insulin resistance. Inflammation in the intestinal mucosa causes a loss of intestinal barrier integrity and increased transport of LPS into the blood by chylomicrons [69]. This process is manifested by endotoxemia and increased production of proinflammatory cytokines (TNF-alpha, IL-1, IL-6) by immune cells as a result of binding of LPS to the CD14/TLR4 complex on their surface. LPS interaction with TLR-4 induces conformational change promoting the recruitment of adapter molecules such as MyD88 protein, IRAK, TRAF6, and NIK to intracellular domain, thereby stimulating the phosphorylation and degradation of IKKB, the NF-κB inhibitors. Translocation of active NF-κB to the nucleus activates the expression of inflammatory proteins and triggers signaling pathways such as JNK, p38 MAPK, and ERK, which induces insulin resistance leading to obesity [71].

Increased production of short chain fatty acids (SCFAs) by gut microbiota provides additional calories to the host, leading to weight gain. They can bind to the G protein-coupled receptor (GPCR) GPR41 (also known as FFAR3) and induce expression of the enteroendocrine hormone peptide YY (PYY) in gut epithelial L-cells, which inhibits gut motility and increases energy harvest from the diet in mice. Moreover, the binding of SCFAs to GPR41 or GPR43 (FFAR2) [72] triggers secretion of the hormone glucagon-like peptide (GLP-1) by intestinal L-cells in mice35, which has a relevant impact on pancreatic function and insulin release, as well as central effects regulating appetite. Feces of obese individuals showed an increased level of SCFAs when compared with lean individuals [73].

Microbiota influences lipoprotein lipase activity by altering the expression of fasting-induced adipose factor (FIAF), the inhibitor of lipoprotein lipase activity causing accumulation of triglycerides (TG) in adipocytes. An increased level of TG in adipose tissue causes hypertrophy, leading to chronic inflammation, preventing further deposition of TG in adipose tissue, and thereby promoting ectopic accumulation of TG in other organs developing insulin resistance.

### 7.7. Gut–Liver Axis

Microbiota dysbiosis alters the gut permeability, enhancing the release of bacteria-derived bioactive molecules in the liver. LPS interacts with TLR4 of the Kupffer cells [70], enhancing the recruitment of MyD88 protein, IRAK, TRAF6, and NIK, which promotes activation of MAPK, JNK, p38, and NF-κB signaling pathways leading to inflammation and insulin resistance, ultimately causing nonalcohol fatty liver (NAFLD). Metabolites such as bile acid, SCFAs, and choline play a vital role in causing NAFLD [74].

### 7.8. Gut–Brain Axis

Neuroactive peptides, lactate, SCFAs, and LPS of gut microbiota activate vagal afferent neurons and gut hormones, leading to alteration in appetite and neuroinflammation [70].

## 8. Conclusions

Obesity is one of the most urgent problems that modern societies face [1]. Taking into consideration the wide range of comorbidities associated with excess body weight, finding the solutions to reduce the magnitude of the problem remains one of the most significant issues for scientists around the world [11,13]. Beyond doubt, many different factors are involved in the etiology of obesity. Changes in the composition of gut microbiota appear to be one of them and therefore have become the subject of detailed research [3].

The bacteria present in the intestines form a complex, multifunctional system that might play a vital role in the maintenance of metabolic balance. It has been proved that alterations in the gut microbiota are found in patients with metabolic diseases (e.g., obesity with associated conditions). The mechanism, in which this relationship occurs, has not been fully explained yet [36,38]. Nevertheless, the studies show that there are a few molecular pathways involved.

The gut microbiota enables the metabolism of dietary fiber into short chain fatty acids (SCFAs), which could be absorbed or excreted in feces. The studies have shown that SCFAs might have a protective effect on the maintenance of proper body weight. It can be achieved by both improving lipid metabolism on a molecular level as well as acting through gut hormones such as incretin, GIP, PYY, GLP-1, and GLP-2. These hormones are involved in the regulation of satiety and glucose metabolism, which might cause alleviation of insulin resistance and limit the development of obesity [53,56]. On the other hand, an excessive amount of SCFAs could reduce this beneficial metabolic effect due to the increase in energy supply, which might promote weight gain [56]. That is why the role of SCFAs in obesity remains controversial.

Moreover, metabolic endotoxemia induced by LPS (a product of Gram-negative bacteria) might result in chronic inflammatory process, which increases adipocyte hyperplasia and proliferation of adipocytes’ precursors. Healthy gut microbiota has the potential to reduce the levels of LPS in the intestines and therefore alleviate chronic low-grade inflammation and associated adipogenesis [63].

Many studies on both animal and human subjects have shown the difference in body weight between organisms with different composition of gut microbiota. The greatest impact was proved in the changes of Bacteroidetes/Firmicutes ratio and participation of *Lactobacillus* [24,67]. However, many other bacterial genres in the gut microbiota seemed to be associated with a tendency to develop obesity [63,68,69]. Furthermore, there was a noticeable difference in the composition of microbiota and the way of delivery [54]. It suggests that whether the child was born via caesarean section or vaginally might have an impact on its microbiota and future tendency to gain weight.

Even though the dependence between the composition of gut microbiota and obesity has not been fully explored yet, it is certain that patients with unbalanced and undifferentiated microbial community are at higher risk of developing obesity and associated comorbidities [69]. Therefore, the gut microbiota plays a vital role in obesity prevalence, taking into consideration all the environmental and lifestyle factors (e.g., diet, antibiotics and probiotics use, way of delivery) that have an impact on alterations in the composition of this complex system.

These conclusions might lead to potential new ways of treatment of metabolic diseases. Both fecal transplant and usage of probiotics could be effective in changing the composition of gut microbiota and hence the prevention of obesity. However, these types of treatment require further research to assess their efficacy and possible practical use [24,75,76].

## Figures and Tables

**Table 1:** Primary and secondary disease-related causes of obesity.

Primary Causes of Obesity	Secondary Causes of Obesity
Genetic causes	Neurologic
Monogenic disorders	Brain injury, brain tumor
Melanocortin-4 receptor mutation	Consequences of cranial irradiation
Leptin and leptin receptor deficiency	Hypothalamic obesity
Prohormone convertase deficiency	Endocrine
BDNF and TrkB insufficiency	Hypothyroidism ^a^
SIM 1 insufficiency	Cushing syndrome
Proopiomelanocortin deficiency	Growth hormone deficiency
Syndromes	Pseudohypoparathyroidism
Prader–Willi	Psychological
Bardet–Biedl	Eating disorders, depression ^b^
Cohen	Drug-induced
Alström	Tricyclic antidepressants, antipsychotics
Beckwith–Wiedemann	Oral contraceptives
Froehlich	Anticonvulsants
Carpenter	Glucocorticoids
	Sulfonylureas
	Glitazones
	Beta-blockers

^a^ Controversial whether hypothyroidism causes obesity or exacerbates obesity. ^b^ Depression associated with overeating or binging [3].

**Table 2:** Comorbidities of obesity.

Comorbidities of Obesity [15]	
**Medical**	**Psychological**
Dyslipidemia [16]	Negative mood [17]
Hypertension [16]	Attention deficit hyperactivity disorder [18]
Type 2 diabetes mellitus [16]	Depression [17]
Steatohepatitis and/or nonalcoholic fatty liver disease [18]	Poor self-esteem [17]
Polycystic ovary syndrome [18]	Eating disorders [18]
Gastro-esophageal reflux disease [18]	Internet addiction [18]
Obstructive sleep apnea [16]	Conduct issues or disorders [18]
Weight-related joint disease [16]	Reduced quality of life [18]
Benign intracranial hypertension [18]	

## Data Availability

The data used in this article are sourced from materials mentioned in the References section.
